# Wound Healing Properties of Plant-Based Hydrogel and Oleogel Formulations in a Rat Scald Burn Model

**DOI:** 10.3390/pharmaceutics17050597

**Published:** 2025-05-01

**Authors:** Oana Janina Roșca, Alexandru Nistor, Georgeta Hermina Coneac, Ioana Viorica Olariu, Ana-Maria Cotan, Roxana Racoviceanu, Elena Rodica Heredea, Adelin Ciudoiu, Gabriela Didea, Camelia Mihaela Lupou, Florin Borcan, Teodora Hoinoiu, Cristina Adriana Dehelean, Lavinia Lia Vlaia, Codruța Marinela Șoica

**Affiliations:** 1 Department of Pharmacology-Pharmacotherapy, Faculty of Pharmacy, Victor Babes University of Medicine and Pharmacy, Eftimie Murgu Square, No. 2, 300041 Timisoara, Romania; oana-janina.rosca@umft.ro (O.J.R.); camelia.lupou@umft.ro (C.M.L.); codrutasoica@umft.ro (C.M.Ș.); 2 Discipline of Clinical Practical Skills, Department I Nursing, Faculty of Medicine, Victor Babes University of Medicine and Pharmacy, 300041 Timisoara, Romania; elena-rodica.heredea@umft.ro (E.R.H.); tstoichitoiu@umft.ro (T.H.); 3 Plastic Surgery Department, University Hospital UZ Brussel, Vrije Universiteit Brussel, 1090 Brussel, Belgium; 4 Department of Pharmaceutical Technology, Formulation and Technology of Drug Research Center, Victor Babes University of Medicine and Pharmacy, 300041 Timisoara, Romania; coneac.georgeta@umft.ro (G.H.C.); olariu.ioana@umft.ro (I.V.O.); mut.anamaria@umft.ro (A.-M.C.); vlaia.lavinia@umft.ro (L.L.V.); 5 Department of Pharmaceutical Chemistry, Faculty of Pharmacy, Victor Babeș University of Medicine and Pharmacy, 300041 Timisoara, Romania; babuta.roxana@umft.ro; 6 Advanced Cardiology and Hemostaseology Research Center, Victor Babes University of Medicine and Pharmacy, No. 2 Eftimie Murgu Square, 300041 Timisoara, Romania; 7 Faculty of General Medicine, Victor Babes University of Medicine and Pharmacy, 300041 Timisoara, Romania; adelin.ciudoiu@student.umft.ro; 8 Eduard Pamfil Psychiatry Clinic, Pius Branzeu County Emergency Clinical Hospital, 300425 Timisoara, Romania; marielena.didea@student.umft.ro; 9 Department of Analytical Chemistry, Victor Babes University of Medicine and Pharmacy, 300041 Timisoara, Romania; fborcan@umft.ro; 10 Research Center for Pharmaco-Toxicological Evaluations, Faculty of Pharmacy, Victor Babes University of Medicine and Pharmacy Timisoara, 300041 Timisoara, Romania; cadehelean@umft.ro

**Keywords:** wound care, bioactive gels, wound inflammation, burn wound treatment, experimental burns, transdermal delivery, plant extracts, hydrogel, oleogel, wound healing, *Boswellia serrata*, *Ocimum basilicum*, *Galium verum*, *Sambucus nigra*, experimental rat burn model, scald injury, topical therapies

## Abstract

**Background:** Scald burns pose significant morbidity, and effective topical treatments remain a clinical priority. Burn injuries pose a significant clinical challenge due to the prolonged inflammation and high infection risk. Traditional treatments focus on moisture retention and infection prevention, but biocompatible formulations such as hydrogels and oleogels offer advantages. Hydrogels hydrate, cool, and promote epidermal regeneration, while oleogels form a lipid barrier that enhances the absorption of lipophilic bioactive compounds. There is an increasing demand for novel topical alternatives that can effectively improve wound healing by modulating the inflammatory cascade, accelerating epithelial and dermal regeneration, and restoring barrier function. **Objective:** This study aimed to determine the most effective plant-based topical formulations for enhancing second-degree scald burn wound healing. **Methods:** Utilizing a standardized rat model, we compared 21 distinct topical formulations, consisting of oleogel and hydrogel bases enriched with extracts from *Boswellia serrata* (frankincense), *Ocimum basilicum* (basil), *Sambucus nigra* flower (elderflower), and *Galium verum* (lady’s bedstraw). Second-degree burns were uniformly induced in 24 Wistar rats using boiling water (100 °C for 8 s) using the RAPID-3D device, a validated 3D-printed tool that ensures reproducible burns through controlled exposure to boiling water. Post-burn, rats were divided into three equal subgroups, and topical formulations were applied daily. Wound healing efficacy was evaluated through wound surface area measurements, transepidermal water loss (TEWL), skin hydration, sebum production, pigmentation, inflammation (erythema), skin perfusion, and histological parameters at multiple timepoints (days 1, 4, 9, 14, and 21 post-burn induction). **Results:** Statistical analyses indicated significant advantages of oleogel-based formulations over hydrogel-based formulations. Specifically, formulations containing *Boswellia serrata* and *Ocimum basilicum* extracts significantly reduced wound size and inflammation, improved skin hydration, and decreased melanin production by days 9 and 21 (*p* < 0.05). **Conclusions:** These findings underscore the potential clinical value of oleogel-based topical preparations containing specific plant extracts for improving scald burn wound healing outcomes, warranting further clinical evaluation.

## 1. Introduction

Burn injuries, particularly scald burns, constitute a critical healthcare concern due to their high morbidity, risk of complications, and substantial impact on patient quality of life. Burn injuries affect over 9 million people annually and cause approximately 120,000 deaths [1], particularly in low- and middle-income countries. These injuries lead to significant physical disfigurement, psychological trauma, and long-term disabilities [2].

Second-degree burns, characterized by damage extending through the epidermis into the superficial dermis, require prompt and effective intervention to prevent complications such as infections, delayed healing, hypertrophic scarring, and long-term functional impairment [3].

Burn wounds trigger an immediate inflammatory cascade, increasing capillary permeability, which leads to protein leakage and interstitial edema [4]. The loss of the epidermal barrier exacerbates trans-epidermal water loss (TEWL), resulting in dehydration [5]. The resulting oxidative stress amplifies the inflammatory damage, further impairing re-epithelialization and delaying wound closure [6,7]. In severe cases, these processes escalate to systemic inflammatory response syndrome (SIRS) and sepsis, increasing mortality risk [8,9]. Additionally, severe burns can induce hypovolemia and reduced cardiac output, a condition known as burn shock, necessitating urgent fluid resuscitation to restore hemodynamic stability [10].

Following the acute phase, burn patients experience prolonged hypermetabolism, chronic inflammation, and lean body mass loss, which impair wound healing and delay re-epithelialization, increasing the risk of complications and extended hospital stays [11].

Topical formulations have long been integral to burn management, providing a barrier against infection, facilitating hydration, and promoting regeneration. Conventional treatments, including silver sulfadiazine (SSD) and antiseptic agents such as povidone-iodine, alongside synthetic dressings to prevent infection and maintain hydration, possess known limitations such as limited efficacy in promoting healing, potential for bacterial resistance, and allergic reactions [12,13]. There is an ongoing need for novel and effective alternatives to these conventional treatments, because SSD delays healing by inhibiting fibroblast proliferation and increasing oxidative stress [12,14], resulting in prolonged recovery and a higher risk of hypertrophic scarring [15,16].

Maintaining a moist environment [17] is crucial for effective wound healing, as it enhances cell migration and re-epithelialization, whereas dry conditions lead to dehydration and scab formation, delaying recovery [18,19]. These limitations underscore the need for alternative therapies that not only prevent infection but also actively promote tissue regeneration.

Recent research highlights the potential of hydrogels and oleogels in optimizing burn wound healing. Hydrogels, composed of a water-rich polymer network, provide hydration, cooling effects, and mechanical protection, making them ideal for early-stage burn management [20]. Their hydrophilic nature enables the incorporation of bioactive compounds, promoting epithelial regeneration and moisture balance. Conversely, oleogels, structured as semi-solid lipid matrices, enhance moisture retention and the transdermal penetration of lipophilic bioactives, offering sustained drug release and protection against oxidative stress [21]. By preserving hydration, modulating inflammation, and enabling targeted drug delivery, these gel-based systems present a promising alternative to conventional burn therapies [22].

Despite progress in hydrogel and oleogel wound dressings, their combination with medicinal plant extracts remains largely unexplored.

Recent research has increasingly focused on plant-derived ingredients for wound healing due to their anti-inflammatory, antimicrobial, and regenerative properties. Ingredients such as *Boswellia serrata* (frankincense), *Ocimum basilicum* (basil), *Sambucus nigra* flower (elderflower), *Sambucus nigra* bark, and *Galium verum* have individually demonstrated therapeutic promise in wound management through various bioactive components. The flavonoids, polyphenols, and terpenoids found in basil, elderberry, frankincense, and lady’s bedstraw possess well-documented anti-inflammatory and antioxidant effects, which may counteract the excessive oxidative stress and prolonged inflammation seen in burn wounds. However, direct comparative evidence evaluating their efficacy in formulated topical preparations, particularly comparing different bases such as oleogels and hydrogels, remains sparse. In previous studies, the potential of oleogel formulations has been highlighted due to their lipid-rich composition, improving skin hydration and active ingredient bioavailability. Conversely, hydrogels have also been favored for their cooling properties and ease of application. To directly assess and compare these benefits, we developed a range of plant-based topical formulations using both oleogel and hydrogel bases and systematically evaluated their effectiveness on standardized burn wounds.

This study investigates hydro- and oleogel formulations enriched with basil, elderflower and elder-bark, frankincense, and lady’s bedstraw, all traditionally used in herbal medicine for their regenerative effects [23,24,25,26]. Anecdotal experience with Romanian traditional medicine formulation, which the first author used to treat an extensive second-degree burn on the hands sustained at age 24, represented the start of our research idea.

In order to investigate whether plant-enriched hydrogel and oleogel formulations can effectively reduce inflammation and enhance wound healing, we employed the RAPID-3D (Rat Printed Induction Device—3D) [27], previously validated in our laboratory, designed to consistently produce uniform second-degree scald burns through controlled exposure to boiling water in Wistar rats.

**Figure 1:**
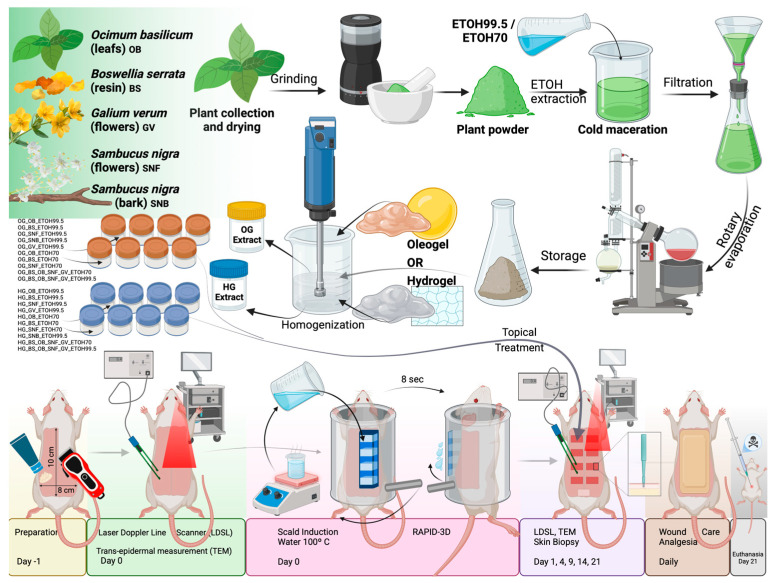
Schematic illustration of the experimental protocol for evaluating topical plant-based oleogel and hydrogel formulations in wound healing. Plant materials were collected, dried, and processed into powders. Active extracts were obtained by cold maceration using ethanol solvents, filtered, concentrated via rotary evaporation, and stored until further use. Extracts were homogenized into oleogel (OG) or hydrogel (HG) formulations. Wounds were uniformly induced on rat dorsal skin using the RAPID-3D device with 100 °C water exposure (8 s). Treatments were applied topically, and healing progress was monitored using a Laser Doppler Line Scanner (LDLS), trans-epidermal measurements (TEM), and skin biopsies collected on days 1, 4, 9, 14, and 21. Daily wound care and analgesia were provided until euthanasia at day 21.

We hypothesized that topical oleogel and hydrogel formulations enriched with specific plant extracts (*Boswellia serrata, Ocimum basilicum, Sambucus nigra* flower and *Sambucus nigra* bark, *Galium verum*, and a combined extract of these four) would significantly enhance second-degree burn wound healing outcomes compared to untreated burns and cream base-only formulations, when used in a Wistar rat scald wound model (see Figure 1).

The primary outcome measure was wound contraction rate. By assessing key wound healing parameters such as the erythema index, TEWL, hydration levels, and histological changes, this study aims to provide scientific validation for these plant-based biocompatible gel formulations as novel burn therapies.

## 2. Materials and Methods

Using an animal scald wound models is crucial for studying the pathophysiological changes associated with this condition and for the development of new treatments for burn injuries [28].

### 2.1. Plants

A total of 21 topical formulations were prepared, including oleogels and hydrogels enriched with the following plant extracts:

- *Boswellia serrata* (BS),
- *Ocimum basilicum* (OB),
- *Sambucus nigra* flower (SNF),
- *Sambucus nigra* bark (SNB),
- *Galium verum* (GV),
- A combined extract containing equal parts by weight of the four plant extracts (BS_OB_SNF_GV).

Plant material was sourced from the following:

- Commercially available tea preparations purchased from an online pharmacy (planteea.ro):
  ○ Basil tea (Ceai de busuioc, Lot 1958, SC Stefmar Productie SRL, Ramnicu Valcea, Romania),
  ○ Lady’s Bedstraw tea (Sânziene galbene, Lot 84941, Dacia Plant, Bod, Romania),
  ○ Elderflower tea, (Soc flori, Lot 85053, Dacia Plant, Bod, Romania),
- Incense granules (100% Tămâie 100%, Lot 5819 LIFE Bio, SC Bionovativ SRL, Podu Olt, Romania),
- Harvested from wild-growing plants (bark from young branches of *Sambucus nigra* bushes of 2 m height, collected from the outer part of the tree).

All plant material was allowed to fully dry in controlled temperature and humidity conditions (22 ± 2 °C; 50 ± 10% RH), after which they were individually ground to a fine powder using an electric grinder.

### 2.2. Plant Extraction Procedure

For each plant, two types of plant extracts were prepared using absolute ethanol (99.5%) and 70% ethanol (EtOH, Chimreactiv, Bucharest, Romania).

The extracts were prepared as follows: (1) the plants were finely ground; (2) 90 g of each herbal material was added to an Erlenmeyer flask, followed by the addition of 900 mL of solvent; (3) the mixture was stirred at 60 °C for 2.5 h; (4) subsequently, the mixture was allowed to cool to room temperature; (5) the sample was first homogenized for 48 h and subsequently subjected to sonication at 40 °C for 1 h; (6) the mixture was then refrigerated at 4 °C for 3 days; and (7) finally, the mixture underwent filtration, and the solvent was removed under vacuum using a rotary evaporator. The dry plant extracts were stored and labeled in air-sealed containers.

### 2.3. Chemical Agents and Excipients

Oleogel and hydrogel formulations of the plant extracts were prepared using either of the following:

- An oleogel base {Olive oil (Ulei de măsline, Azelis SRL, Bucharest, Romania) 80% *w*/*w* OR sunflower oil (Ulei de floarea soarelui, Azelis SRL, Bucharest, Romania) 80% *w*/*w* OR Isopropyl myristate (Sigma-Aldrich, Taufkirchen, Germany) 80% *w*/*w* OR Diethylene glycol monoethyl ether (Thermo Fisher Scientific, Bremen, Germany) 80% *w*/*w* AND Glyceryl dibehenate (Compritol 888 ATO, Gattefossé, Saint-Priest Cedex France) 20% *w*/*w*},
- A hydrogel base {purified water 65% *w*/*w*, Poloxamer 407 (Kolliphor^®^ P 407 Geismar, BASF Pharma, Hannover, Germany) 25% *w*/*w*, and glycerol 10% *w*/*w*}.

All other materials were of analytical purity and were used as received. Topical formulations did not contain microbial stabilizers and maintained their rheological properties throughout this study.

Plant extracts were mixed with either the oleogel or the hydrogel base, generating a total of 20 topical formulations, as described in Table 1. The *Sambucus nigra* bark extracted in 70% ethanol and *Galium verum* extracted in 70% ethanol could not be incorporated into the single-plant topical formulations.

### 2.4. Preparation of Blank and Plant Extract-Loaded Oleogel Formulations

The plant extract-loaded oleogels were made by mixing Compritol 888 ATO with oily liquids (diethylene glycol monoethyl ether, isopropyl myristate, sunflower oil, olive oil) and heating the mixture to 80 °C while stirring (600 rpm) until a clear, uniform oily liquid was produced. At room temperature, stirring persisted until the mixture reached 50 °C, at which point, the plant extract—described in Table 1—was added at 5% (*w*/*w*). After cooling to room temperature, the final homogenous mixture was formed into the oleogel. A simple oleogel formulation devoid of the plant extract was generated using the same technique. The decision to use a 5% concentration of plant extract in the formulations was guided by the existing literature, indicating that a 5% concentration is adequate and appropriate for topical application [23,29,30,31].

### 2.5. Preparation of Blank and Plant Extract-Loaded Hydrogels Based on Poloxamer 407

Poloxamer-based hydrogel formulations were prepared with plant extract using the cold method [32]. The control poloxamer-based hydrogel was prepared using the following steps: Poloxamer 407 was added to purified water and cooled to 4 °C. The mixture was refrigerated for at least 24 h with periodic stirring until a clear, homogeneous solution was obtained. For plant extract-loaded hydrogels, the plant extract and glycerol were added to the cold poloxamer solution under continuous stirring until its standard characteristics were obtained. The plant extracts and excipients used are specified in Table 1.

### 2.6. Animals

Thirty female Wistar rats weighing between 250 and 300 g (average 278 g) were obtained from the animal laboratory of Iuliu Hatieganu University of Medicine and Pharmacy, Cluj-Napoca. The rats were individually housed in well-ventilated cages with highly absorbent bedding that was changed daily. The cages were sanitized prior to the study to prevent infections. Animals had free access to water and solid rodent chow and were maintained under standard intermittent 12 h light/dark cycles in a temperature- and humidity-controlled environment (23 ± 1 °C, 45–50% humidity). The rats were acclimatized to these conditions for one week before the experiment in well-ventilated cages (Makrolon^®^ Type IV, Carfil Quality, Oud-Turnhout, Belgium) [33], lined with wood shavings (Premiumspan small pets, Brandernburg, Germany) and odorless absorbent cage liners (Bamboo Carbon, Paw Inspired^®^, FL, USA), which were changed daily during the cleansing of the cages, in order to avoid adhesion of any particles to the scald wounds. Animals had free access (ad libitum) to food until 12 h before scald induction. During the follow-up period, they were housed with standard intermittent light and dark cycles in a temperature- and humidity-controlled environment (22 ± 2 °C; 50 ± 10% RH) at the “Pius Brânzeu” Laparoscopy and Microsurgery Center, Timisoara. The rats were habituated to handling to reduce stress, and a low-volume radio minimized ambient noise.

All procedures adhered strictly to the ARRIVE guidelines [34] and were approved by the Ethics Committee of Victor Babes University of Medicine and Pharmacy, Timisoara (approval no. 17/11.03.2022).

### 2.7. Anesthesia, Preoperative Preparation, and Pain Management

Prior to inducing burns, the rats were subjected to a 12 h fasting period, during which they had free access to water. Light sedation was achieved using 2% isoflurane while the rats were positioned on a heating pad. Anesthesia was administered via an intraperitoneal injection of a ketamine (100 mg/mL) and xylazine (20 mg/mL) mixture, dosed at 90 mg/kg for ketamine and 10 mg/kg for xylazine. The effectiveness of the anesthesia was verified by pinching one of the limbs. Anesthesia intensity was monitored by assessing the righting and pedal withdrawal reflexes at 7, 10, and 15 min post-induction, with the absence of limb and head reflexes allowing for continued procedures. The hair on the animals’ backs was trimmed in a space the size of 10 cm by 8 cm, starting below the neck and extending down to the iliac bone while also going laterally to the anterior costal margins. To remove any remaining hair, depilatory cream (Veet, Reckitt GmbH, Heidelberg, Germany) was applied to the trimmed area. The area was then disinfected with a povidone-iodine solution and rinsed with sterile water. Basic vital signs were monitored by placing a pulse oximeter on the rat’s tail, connected to a multi-parameter monitor (BM 5, BIO-NET, Berlin, Germany). After burn induction, post-procedure analgesia was provided via subcutaneous butorphanol (0.01–0.05 mg/kg) every 12 h. An Elisabeth cervical collar was placed after scald induction, along with cutting and filing the nails to avoid scratching. The rats were placed post-burn induction on a heated plate (CMA/150 Temperature Controller, CMA/Microdialysis) with real-time intrarectal monitoring, and the plate temperature was maintained at 36.5 °C to avoid post-anesthetic hypothermia and hair loss. Subsequently, they were placed on clean bedding and monitored until they fully recovered from anesthesia. The Parkland formula was used to calculate crystalloid fluid requirements for burn resuscitation in the first 24 h, which were administered intraperitoneally. Post-anesthetic hypersalivation was checked every 30 min using a sterile 5 mL syringe and a 24 G (19 mm) catheter. Throughout the experiment follow-up, the animals were provided free access to water and solid rat chow (ad libitum). Strict aseptic procedures were followed to prevent the transmission of infections, and the animals were routinely monitored for any signs of distress. Humane euthanasia was performed 21 days after the induction of the scald wounds, under full anesthesia, using 0.3 mL/kg of T61 (MSD Animal Health, Intervet International B.V., Boxmeer, The Netherlands). The experimental animals were disposed of as medical waste.

### 2.8. Burn Induction and Wound Standardization

Burn wounds were uniformly induced as previously described using the RAPID-3D device, a validated 3D-printed apparatus designed to provide controlled and reproducible scald burns. Burns were induced by pouring 40 mL of boiling water (98–99 °C) for 8 s onto defined skin areas in the left and right thoraco-dorsal region of the rats. This produced eight consistent second-degree burns, consisting of four symmetrical rectangular scald wounds on each hemi-thorax area, each measuring 20 × 10 mm and spaced 10 mm apart. The Meeh formula was used (TBSA = kW^2/3^, where k = 9.83 and W = weight in kg) to accurately calculate the total body surface area (TBSA), revealing a consistent burn of 4% TBSA across all experimental animals.

In the rat subgroups, an untreated burn area served as the control. An additional unburned skin area served as the normal skin control. The rats were organized into three subgroups of ten animals each, ensuring each formulation was applied consistently across the cohort.

### 2.9. Treatment Application and Dressing Protocol

**Figure 2:**
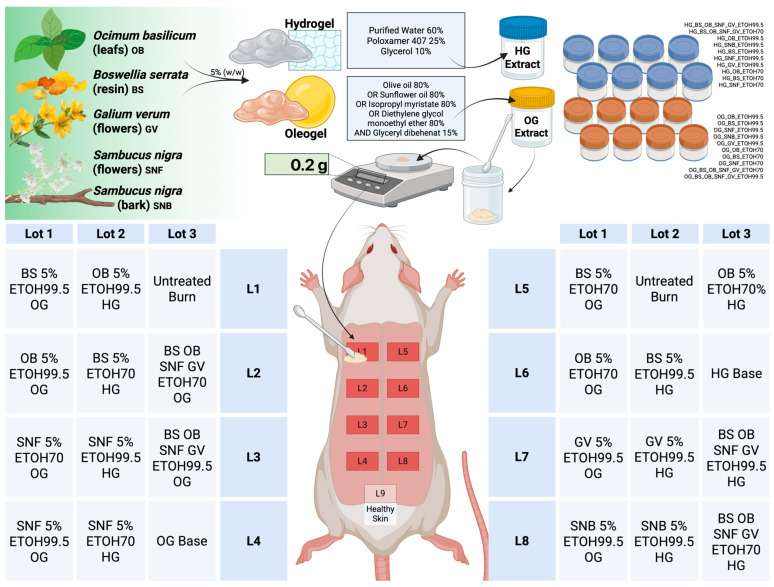
Experimental design and topical treatment allocation for evaluating hydrogel (HG) and oleogel (OG) formulations in a rat scald burn wound healing model. Formulations containing 5% (*w*/*w*) extracts from *Ocimum basilicum* (OB), *Boswellia serrata* (BS), *Galium verum* (GV), *Sambucus nigra* flower (SNF), or bark (SNB) were prepared using either ethanol 70% or 99.5% extraction methods. Each rat received eight distinct treatments (four per side, labeled L1–L8), including single or combined plant extracts in either hydrogel or oleogel vehicles. Control treatments included formulation bases without extracts, untreated burn wounds, and healthy skin (L9) for comparative analysis. The detailed composition and specific formulation for each treatment lot are indicated.

Rats were divided into 3 subgroups of ten rats each. Each subgroup received a different combination of topical treatment using the previously prepared plant extract creams, as depicted in Figure 2, with either an oleogel or a hydrogel matrix. Each burn site was treated with 0.2 g of the assigned hydrogel or oleogel formulation and applied using a sterile microspatula. In each experimental subgroup, scald wounds were left untreated and served as self-controls for the wound healing. Scald wounds treated with blank oleogels and hydrogels, which were devoid of plant extracts, were used as baseline controls. A healthy shaved skin area towards the rat’s tail, which was not burned, served as the healthy skin baseline control. Wounds were then covered with a semi-occlusive dressing, which was changed daily. Before reapplication, residual cream was gently removed with a sterile swab moistened with 0.9% saline, followed by a meticulous drying process with sterile gauze, ensuring the wound is in the best possible condition for the next application. Throughout the experiment duration, the topical formulations were kept refrigerated at 4 °C.

### 2.10. Scald Wounds and Skin and Inflammation Monitoring

To evaluate the progression of burn wound healing, macroscopic evaluation and standardized digital planimetry were used to measure the wound surface area at days 1, 4, 9, 14, and 21 post-burn. A millimeter scale was used to measure each burn location’s length and width, and the burn surface area was calculated in mm^2^.

### 2.11. Skin Perfusion Measurement

The Moor Laser Doppler Laser Scanner (Moor LDLS, Moor Instruments, Devon, UK) assesses skin perfusion by measuring blood flow based on the average speed and concentration of red blood cells. The results are reported in perfusion units (PU), calculated using the first moment of the power spectral density, which ranges from 0 to 500 PU with an accuracy of 3%. This device employs an infrared laser beam (785 nm) to carry out sweeping scans of superficial tissues, yielding images with a resolution of 256 × 256 pixels and a scanning speed of 4 ms per pixel. A baseline measurement was established for each experimental animal before any treatment, followed by scans taken on days 0, 4, 9, 14, and 21 after scald injury induction. Five repeated measurements were taken per wound site in a quiet environment at 24 ± 2 °C.

### 2.12. Transepidermal Skin Biophysical Assessments

At 40 min post-burn, a Multi-Probe Adapter (MPA, Cour-age-Khazaka Electronic GmbH, Köln, Germany) was used to evaluate the following:

- Erythema index and melanin content (Mexameter^®^ MX18),
- Trans-epidermal water loss (TEWL, Tewameter^®^ TM300),
- Skin hydration (Corneometer^®^ CM825),
- Sebum levels (Sebumeter^®^),
- Skin temperature (Temperature Probe).

### 2.13. Histological Evaluation of Burn Wounds

Tissue specimens were obtained using a 2 mm diameter punch biopsy (ZetMedical^®^ Arad, Romania) on days 1, 4, 9, 14, and 21 under light sedation using 2% isoflurane, with the rats positioned on a heating pad. Histological analyses were performed on biopsies stained with hematoxylin and eosin (HE) and Masson’s trichrome. Granulation, fibrosis, inflammation, and epithelization were independently scored on a 0–4 scale by a blinded pathologist. To evaluate inflammation, fibroblast proliferation, angiogenesis, and collagen deposition, tissue sections were stained with hematoxylin–eosin (H&E) and Masson’s trichrome.

### 2.14. Euthanasia

At 21 days, rats were euthanized under full anesthesia, using 0.3 mL/kg T61 (MSD Animal Health, Intervet International B.V., Boxmeer, The Netherlands). Experimental animals were disposed of as medical waste.

### 2.15. Statistical Analysis

A power analysis (α = 0.05, β = 0.80) determined that N = 10 rats was sufficient for each of the three subgroups that received the same topical formulation treatment to the eight scald wounds. No recorded data were excluded. Paired *t*-tests were used to compare burned vs. controls within each animal. One-way ANOVA followed by Tukey’s Honestly Significant Difference (HSD) post hoc tests was used to determine significant differences between formulations. Pearson’s correlation coefficient was used to assess the relationships between vascular damage and histological severity. Statistical analyses were performed using GraphPad Prism 10, with *p* < 0.05 considered significant.

## 3. Results

**Figure 3:**
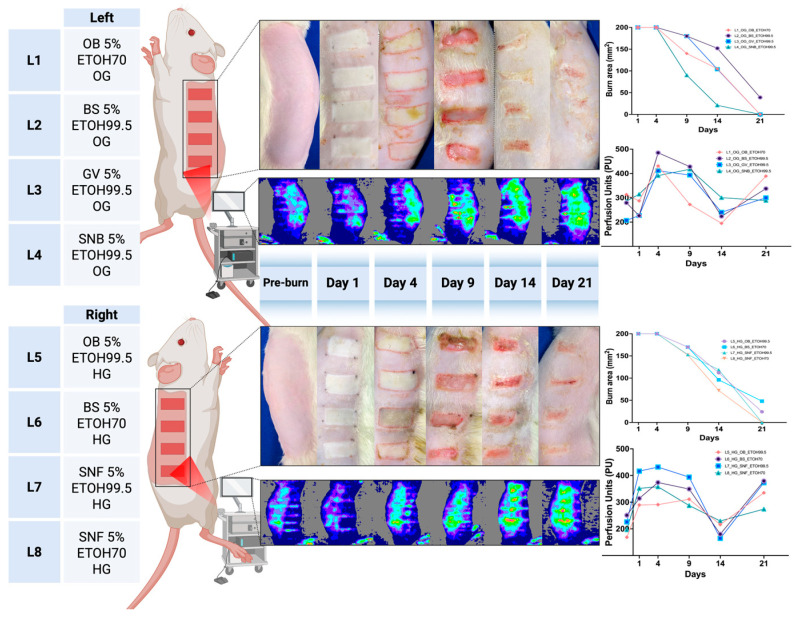
Representative digital planimetry and perfusion imaging outcomes of scald burn wound healing in rats treated with oleogel (OG, top panel) and hydrogel (HG, bottom panel) formulations containing 5% plant extracts. We exemplify a rat with four different topical treatments applied on each dorsal side (L1–L4: oleogel; L5–L8: hydrogel), including extracts from *Ocimum basilicum* (OB), *Boswellia serrata* (BS), *Galium verum* (GV), and *Sambucus nigra* flower (SNF) or bark (SNB), prepared using ethanol solvents (70% or 99.5%). Sequential photographs and corresponding Laser Doppler Line Scanner (LDLS) perfusion images illustrate wound progression at days 1, 4, 9, 14, and 21 post-injury. Graphs demonstrate quantitative changes in burn area (mm^2^) and skin perfusion (perfusion units, PU), highlighting differences in healing dynamics between hydrogel- and oleogel-based formulations.

Representative outcomes of burn wound healing using oleogel and hydrogel formulations are presented in Figure 3. The photographic and laser Doppler perfusion images illustrate clear differences in the healing dynamics between formulations containing different plant extracts. Oleogel treatments generally showed accelerated wound contraction and improved skin perfusion compared to hydrogel treatments, particularly evident from day 9 onwards.

### 3.1. Wound Surface Area Reduction

Quantitative analysis revealed notable differences between the treatment groups. At day 4 post-induction, oleogel formulations showed an average wound surface reduction to 148 mm^2^, while hydrogel-treated wounds were 165 mm^2^. By day 9, oleogel formulations exhibited further reduced wound sizes to an average of 78 mm^2^, compared to the 145 mm^2^ observed with hydrogel treatments. By this study’s conclusion (day 21), oleogel treatments achieved an average wound size of 10 mm^2^, in contrast to 20 mm^2^ for hydrogels and 25 mm^2^ for untreated burns.

At day 9, ANOVA revealed significant differences among the topical formulations (F = 1.92, *p* = 0.035). At day 14, ANOVA only approached significance (F = 1.67, *p* = 0.077) but with no significant pairwise differences. When the formulations were grouped, oleogels enriched with plant extracts showed significantly better wound size reduction compared to hydrogels at day 9 (mean difference: −60.89 mm^2^, *p* = 0.0013) and day 14 (mean difference: −40.70 mm^2^, *p* = 0.0299). Clinically, a discrete erythematous halo was observed after 14 days in the scald wounds treated using oleogel with *Boswellia serrata* extract in 70% ethanol.

**Figure 4:**
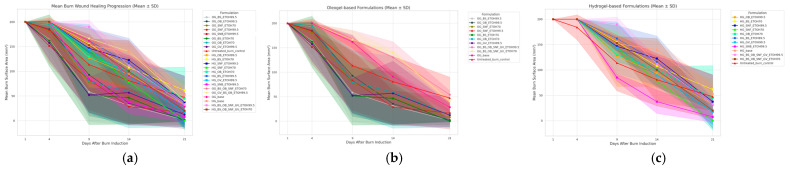
Mean burn wound healing progression (burn surface area in mm^2^) over a 21-day period comparing different topical formulations. (**a**) Combined plot summarizing all formulations; (**b**) healing progression in oleogel formulations; (**c**) wound healing progression in hydrogel formulations. Shaded areas represent ± standard deviation (SD). Each graph shows mean values ± standard deviation (SD) comparing hydrogel, oleogel, base formulations, and untreated burn controls (red).

The overall progression of burn wound healing across all treatment groups is summarized quantitatively in Figure 4a. The mean burn surface area reduction over the 21-day treatment period illustrates notable differences among oleogel and hydrogel formulations containing various plant extracts, as well as formulation bases and untreated controls. Oleogel-based formulations (see Figure 4b) consistently demonstrated enhanced wound area reduction compared to hydrogels (see Figure 4c) and controls, particularly from days 9 to 21. Among individual plant extracts, oleogels containing *Boswellia serrata* (BS) and *Galium verum* (GV) exhibited faster and more consistent healing outcomes.

### 3.2. Transepidermal Water Loss (TEWL) and Skin Hydration

**Figure 5:**
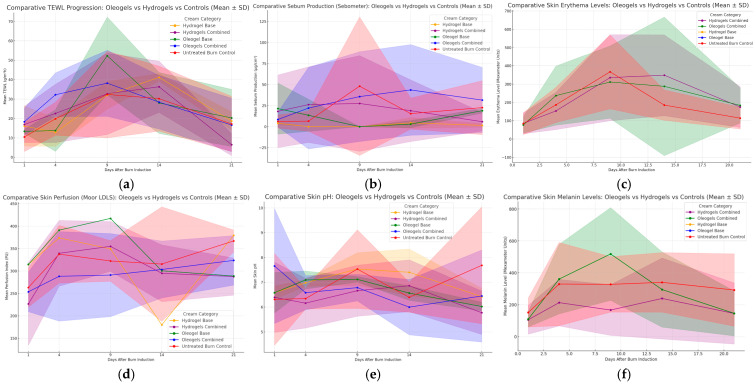
Comparative evaluation of skin parameters during burn wound healing using oleogel and hydrogel formulations versus controls over a 21-day period. Parameters assessed include (**a**) trans-epidermal water loss (TEWL, g/h/m^2^); (**b**) sebum production (μg/cm^2^); (**c**) skin erythema levels (arbitrary Mexameter units); (**d**) skin perfusion measured by Moor Laser Doppler Line Scanner (perfusion units, PU); (**e**) skin pH; and (**f**) skin melanin content (Mexameter units). Each graph shows mean values ± standard deviation (SD) displayed as shaded areas and compares hydrogel, oleogel, base formulations, and untreated burn controls (red).

Oleogel formulations consistently improved skin barrier function more effectively. On day 9, the average TEWL values (see Figure 5a) for oleogels and their base formulation were 32 g/m^2^h and 50 g/m^2^h, respectively, compared to 42 g/m^2^h for hydrogels and 47 g/m^2^h for untreated controls. Nevertheless, these differences were not statistically significant (day 9 F = 7.76, *p* = 0.996). By day 21, all treated groups approached similar TEWL values (~15 g/m^2^h), significantly outperforming the untreated controls (average 23 g/m^2^h). At day 21, significant differences emerged in skin hydration as measured by the Corneometer (F = 2.35, *p* = 0.0062), with oleogel formulations OG_BS_ETOH70, OG_OB_ETOH70, and OG_OB_ETOH99.5 showing significantly higher hydration than hydrogel formulations (*p* < 0.05).

### 3.3. Sebum Production (Sebometer)

Sebum production remained stable across treatments, though oleogels maintained a balanced sebum level (~25 μg/cm^2^) throughout the observation period, whereas hydrogel-treated and untreated wounds showed greater variability (see Figure 5b). On day 9, untreated burns exhibited a notable peak (~50 μg/cm^2^), reflecting barrier disruption, whereas the treated groups remained stable. No statistical significance was achieved between formulations at day 9 (F = 30.45, *p* = 0.083).

### 3.4. Mexameter (Melanin and Erythema)

Erythema values declined faster with oleogel treatments (see Figure 5c), reaching an average of 200 units by day 14, compared to 275 units for hydrogels and 300 units for untreated burns. Melanin levels did not differ significantly between treatments (see Figure 5f), remaining relatively consistent (~400 units). At day 9, significant differences in melanin levels (Mexameter) were observed (F = 39.74, *p* < 0.001). Post hoc analysis showed that oleogel formulation OG_BS_ETOH70 significantly outperformed several hydrogel formulations (*p*-values ranging from 0.0118 to 0.0404). At day 21, both melanin (F = 5.11, *p* < 0.001) and erythema (F = 5.92, *p* < 0.001) showed significant differences, with oleogel formulations OG_OB_ETOH70 and OG_OB_ETOH99.5 consistently displaying significantly lower values compared to hydrogel formulations (*p* < 0.05).

### 3.5. Skin pH

The skin pH of the treated scald wounds varied across the individual formulations throughout this study (see Figure 5e). Oleogels normalized skin pH more effectively, achieving near-optimal values (~6.5) by day 14. Hydrogels demonstrated a slower normalization, achieving approximately pH 7 by day 14. Untreated controls showed persistent elevations (pH ~7.5–8), indicative of slower recovery. At day 14, skin pH demonstrated significant overall differences (F = 1.99, *p* = 0.0083), but no individual formulation differences reached statistical significance upon pairwise comparisons.

### 3.6. Skin Perfusion (Moor LDLS)

Skin perfusion analysis indicated initial perfusion increases peaking around day 4 (~400 PU for oleogel base) that then progressively normalized (see Figure 5d). Oleogels consistently provided better perfusion enhancement compared to hydrogels throughout the study period, with hydrogel formulations closely matching untreated controls by day 21 (~350 PU). Despite this, no statistically significant differences were observed among formulations regarding skin perfusion measured by Moor LDLS at days 9 (F = 0.87, *p* = 0.562), 14 (F = 0.65, *p* = 0.935), or 21 (F = 1.23, *p* = 0.137).

### 3.7. Skin Hydration (Corneometer)

Corneometer data indicated significantly higher hydration for oleogel treatments, averaging around 45 units by day 14 compared to hydrogels at approximately 40 units. The untreated control consistently showed the lowest hydration levels (~30 units). By day 21, oleogel treatments reached near-normal hydration (~50 units), whereas hydrogels lagged slightly behind (~45 units).

### 3.8. Histological Evaluation

**Figure 6:**
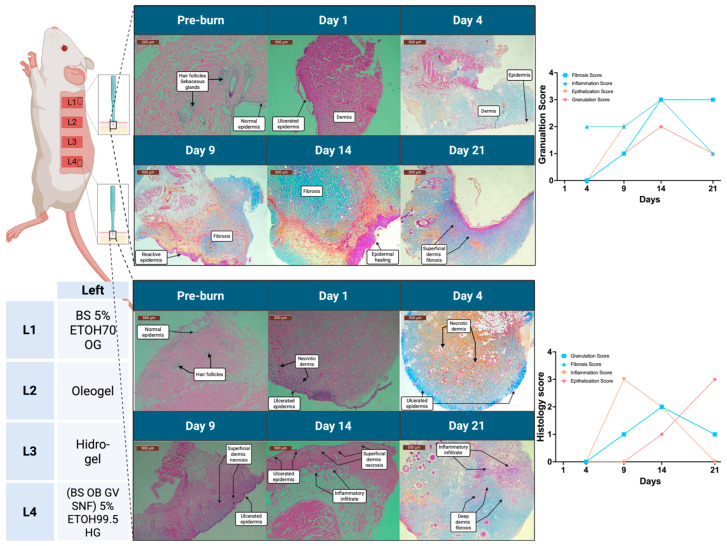
Representative histological evaluation of scald burn wound healing progression in rats treated with oleogel and hydrogel formulations. Skin biopsies stained with Masson’s trichrome illustrate histological changes at pre-burn and days 1, 4, 9, 14, and 21 post-injury. The upper panel shows healing with *Boswellia serrata* (BS) 5% ethanol 70% oleogel formulation; the lower panel illustrates healing progression with a combination hydrogel formulation containing 5% plant extracts of *Boswellia serrata* (BS), *Ocimum basilicum* (OB), *Galium verum* (GV), and *Sambucus nigra* flower (SNF) in ethanol 99.5%. Graphs depict semi-quantitative histology scoring of fibrosis, inflammation, epithelialization, and granulation tissue formation on a 4-point scale, reflecting differential effects of formulations on tissue remodeling and wound healing dynamics. Scale bars represent 200 μm (pre-burn) and 500 μm (days 1–21).

**Figure 7:**
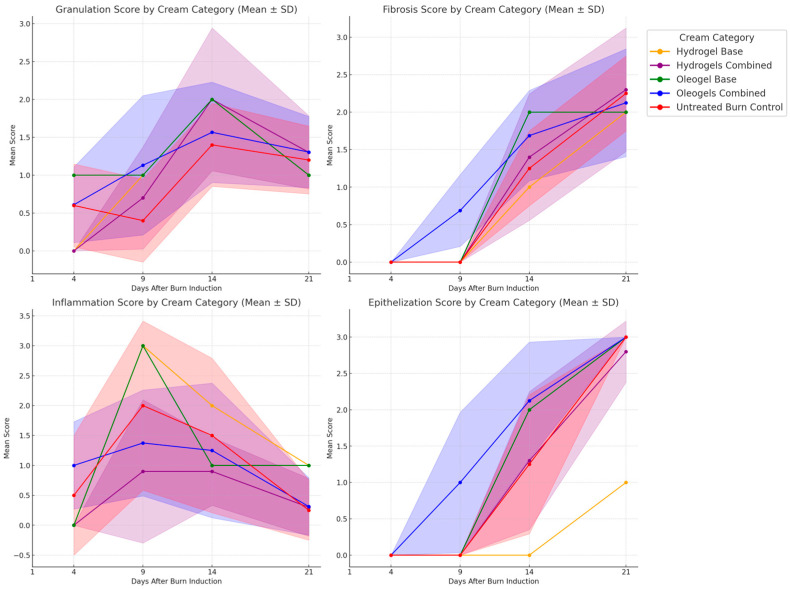
Comparative histological evaluation of burn wound healing parameters (granulation tissue, fibrosis, inflammation, and epithelialization). Mean scores (±SD, displayed as shaded areas) over the 21-day observation period are presented for all hydrogels combined (purple), oleogels combined (blue), respective formulation bases (orange and green), and untreated burn controls (red). Higher scores reflect greater levels of granulation tissue formation, fibrosis, inflammation, or epithelialization, indicating the differential effects of formulation type on the inflammatory response during burn healing.

Histological observations from representative biopsies illustrate the healing dynamics induced by specific topical formulations, as shown in Figure 6. Treatment with *Boswellia serrata* (BS) 5% oleogel exhibited pronounced granulation tissue formation and effective epithelialization by day 14, coupled with a controlled inflammatory response. In contrast, the combined hydrogel formulation containing BS, OB, GV, and SNF showed a more prolonged inflammatory phase, accompanied by delayed granulation and epithelialization. Scores for granulation, fibrosis, inflammation, and epithelialization were assessed on a 4-point scale and indicated clear formulation-dependent differences (see Figure 7). Histological scoring demonstrated enhanced wound healing quality with oleogel treatments, as evidenced by the lower inflammation scores (~0.5 by day 14), higher epithelization scores (~2.5 by day 21), improved granulation (~2.0 by day 14), and reduced fibrosis (~1.5 by day 21). Hydrogel-treated wounds showed improvement over untreated controls but consistently scored lower compared to oleogels. Histologically, microbial colonies were observed developing on the blank hydrogel-treated scald wounds. No such colonies were observed in other topical formulations with plant extracts or the blank oleogel control-treated wound. SNF Et-OH 70% and SNB Et-OH 99.5% showed giant multinucleated foreign bodies on H&E slides.

Overall, these results demonstrate the consistent advantages of oleogel-based formulations containing *Boswellia serrata* and *Ocimum basilicum* extracts in promoting effective wound healing, reducing inflammation, and improving skin hydration and pigmentation outcomes.

## 4. Discussion

### 4.1. Overview of Standard Burn Care and Limitations of SSD

Second-degree burns, also known as partial-thickness burns, affect both the epidermis and the dermis layers of the skin. Traditional treatments aim to alleviate pain, prevent infection, and promote healing. Standard care often includes cleaning the wound, applying antibiotic ointments, and using dressings to protect the area. In some cases, especially with extensive burns, skin grafting may be necessary to facilitate recovery [35].

Considering the remarkable progress in biotechnological research, additional investigation is warranted to achieve a better knowledge of the interplay between the compositions of gel varieties, active phytoconstituents, and the skin, thus enhancing the efficacy of individual burn therapies [36].

Developing formulations that are safe for both the patient and the environment poses several challenges. These include the complex behavior of oleogels or hydrogels in various solvents, with limited knowledge about their interaction with different constituents and how bioactive compounds interact under different processing conditions.

### 4.2. Comparative Analysis of Wound Healing Effects of Studied Plant Extracts

Recent research has investigated the use of hydrogels in skin grafting and clinical burn care. A single center randomized controlled trial (RCT) showed that wound gel dressings are a good substitute for traditional treatments and are better than Flammazine in terms of wound assessment (*p* = 0.002), staining (*p* = 0.007), leakage (*p* = 0.032), and odor (*p* = 0.034). However, Flammazine showed better results in terms of dressing dehydration parameters (*p* = 0.012), wound adhesion (*p* = 0.005), and pain when the dressing was removed [37]. In a prospective RCT, hydrogels were not better than traditional plasticized polyvinyl chloride film in terms of providing efficient analgesia and quicker recovery [38]. In contrast, healing time was significantly shorter with liposome polyvinyl-pyrrolidone-iodine hydrogel (PVP-I hydrogel) (9.9 ± 4.5 days) than with silver sulfadiazine (SSD) (11.3 ± 4.9 days, *p* < 0.015) [39]. As a result, poloxamer-based hydrogels might be a substitute for the conventional treatment of iodine-based ointments or Flammazine, which can be harmful to the skin due to their antiseptic properties [29]. SSD cream has been a mainstay in the prevention and treatment of wound infections for patients with second- and third-degree burns [40]. However, SSD may impede the wound healing process, as wounds treated with SSD experienced delayed healing compared to those treated with saline-soaked dressings [33]. In a clinical trial involving 177 children with partial-thickness burns, those treated with SSD had a significantly longer time to complete healing and an increased requirement for compression garments for scar therapy compared to topical antimicrobial (TA) ointment [15].

SSD dressings provided a shorter mean time to re-epithelialization (6.2 +/− 2.8 days) compared to petrolatum gel (7.8 +/− 2.1 days) (*p* = 0.050) in patients with superficial partial-thickness burns [41]. Wound healing was significantly greater for Oleogel-S10^®^ containing birch bark and sunflower oil (85.7%, n = 30) compared to the wound gel Octenilin^®^ (Octenidine + phenoxyethanol) (14.3%, n = 5, *p* < 0.0001) [42].

These findings highlight the need for alternative treatments that prevent infection and promote faster or the same wound healing. Hydrogels and oleogels have emerged as promising candidates in this regard. Their biocompatibility and ability to maintain a moist wound environment can enhance healing outcomes, potentially addressing the limitations associated with traditional SSD therapy. Hydrogels are hydrophilic polymer networks that maintain a moist environment conducive to healing, while oleogels provide a hydrophobic matrix that can serve as a barrier against external contaminants. Incorporating medicinal plant extracts into these gels has been a focus of recent research, aiming to enhance their therapeutic efficacy [15,29,30,32,34,35].

### 4.3. Plant-Derived Bioactive Hydrogels and Oleogels in Burn Wound Healing

Basil (*Ocimum basilicum*) is renowned for its anti-inflammatory, antimicrobial, and antioxidant properties. A study evaluating a hydrogel based on *Ocimum basilicum* and *Trifolium pratense* (red clover) extracts demonstrated significant wound healing effects both in vitro and in vivo, suggesting potential benefits in burn treatment [23]. Furthermore, an aqueous extract-based ointment of *Ocimum basilicum* has been shown to enhance wound contraction, increase hydroxyproline levels, and modulate inflammation, supporting its bioactive role in cutaneous repair [43].

Elderflower (*Sambucus nigra)* possesses anti-inflammatory and antioxidant activities, which may contribute to wound healing. While specific studies on its application in burn treatment are limited, its traditional use in skin ailments indicates potential benefits. Furthermore, PLGA and PCL-based nanoformulations of *S. nigra* extracts have been shown to enhance collagenase inhibition and prolonged anti-inflammatory effects, which may improve burn wound healing outcomes [44]. To our knowledge, limited research has been performed on *Sambucus nigra* bark involving “in vivo” testing.

Frankincense (*Boswellia serrata*) is known for its anti-inflammatory properties attributed to boswellic acids. These compounds have been shown to modulate inflammatory responses, which is crucial in wound healing [45]. A study conducted on Wistar rats found that an oleogel containing *Boswellia serrata* oleo-gum resin at a concentration of 15% had a significantly higher wound contraction rate than povidone-iodine and performed satisfactorily at 10% and 5% concentrations [31].

Lady’s bedstraw (*Galium verum*) is traditionally used for its wound healing properties and has shown antibacterial and antioxidant effects [46], though specific studies focusing on its efficacy in burn treatment remain scarce. A study on a mucoadhesive oral gel containing *Galium verum* indicated enhanced antimicrobial properties and improved wound closure rates [47], making it a potential candidate for burn wound dressings.

Table 2 compares the existing literature on plant formulations using these four active plant extracts.

### 4.4. Experimental Results on Wistar Rats with Scald Wounds Treated with Oleogels and Hydrogels

The present study systematically evaluated a variety of plant-based topical formulations on second-degree scald wounds, demonstrating significant therapeutic advantages for specific oleogel-based preparations. Our results revealed that oleogel formulations enriched with *Boswellia serrata* and *Ocimum basilicum* extracts (OG_BS_ETOH70, OG_OB_ETOH70, and OG_OB_ETOH99.5) consistently outperformed hydrogel-based formulations in wound healing efficacy.

Oleogel-based formulations significantly reduced the wound surface area compared to hydrogel-based treatments, notably at critical healing phases (days 9 and 14), suggesting accelerated re-epithelialization and more effective granulation tissue formation. These findings are consistent with the previous literature highlighting the enhanced hydrating and protective barrier properties of lipid-rich oleogel bases, promoting an optimal wound healing environment. The *Boswellia serrata* oleogel (OG_BS_ETOH70) demonstrated significant acceleration in wound closure, aligning with previous findings where *Boswellia serrata* enhanced fibroblast proliferation and collagen synthesis [39]. Treating scald wounds with *Ocimum basilicum* hydrogels (OG_OB_ETOH70 and OG_OB_ETOH99.5) resulted in superior epithelialization and fibroblast migration, reinforcing its antioxidant-mediated wound healing role.

Skin hydration was significantly improved in oleogel-treated wounds, particularly evident at day 21. Oleogel formulations provided substantially greater moisture retention, essential for promoting effective epithelial regeneration and reducing scarring. These results reinforce existing evidence on the superior barrier properties of oleogels compared to hydrogels.

Pigmentation and inflammation parameters measured by Mexameter indicated significant advantages for oleogel formulations, with OG_BS_ETOH70 notably reducing melanin levels at day 9 and both OG_OB_ETOH70 and OG_OB_ETOH99.5 substantially reducing erythema and melanin at day 21. Reduced melanin and erythema levels suggest lower inflammation and improved cosmetic outcomes, critical factors for patient quality of life post-burn recovery.

While TEWL measurements did not reveal significant differences between formulations, consistent improvements in other biophysical parameters confirm the clinical relevance of oleogel formulations. In contrast, skin perfusion as measured by the Moor LDLS did not significantly differ across treatment groups, possibly indicating that perfusion recovery may require longer observation periods or alternative assessment methods to detect subtle differences.

*Sambucus nigra* extract treatment correlated with marked inflammatory modulation, but its effect on collagen synthesis was less pronounced than *Boswellia serrata*. *Galium verum* hydrogel displayed enhanced antibacterial properties, suggesting it may be more effective in preventing secondary infections in burn wounds. Histological evaluation showed no statistically significant differences between formulations across the evaluated parameters, suggesting that improvements observed clinically may precede clear histological differentiation. This underscores the importance of combining clinical, biophysical, and histological assessments for a comprehensive understanding of topical formulation efficacy.

Wounds treated with the oleogel containing *Boswellia serrata* extract in 70% ethanol exhibited a discrete erythematous halo surrounding the scald lesions. Certain bioactive compounds found in *B. serrata*, such as boswellic acids, although predominantly recognized for their anti-inflammatory properties, might paradoxically induce mild transient hyperemia or vasodilation in sensitive or damaged skin tissue. Alternatively, the halo could represent a subtle hypersensitivity reaction to components within the extract or the vehicle itself; however, such reactions typically present more diffusely rather than as a sharply defined halo. The composition of these extracts has been thoroughly studied and characterized as part of a larger investigation aimed at assessing the wound healing efficacy of four plant-based formulations.

### 4.5. Limitations and Future Directions

This study presents clear strengths, including the standardized and reproducible burn model achieved by the RAPID-3D device, allowing precise comparative evaluations of multiple formulations. However, several limitations must be acknowledged, notably the animal model’s inherent differences from human skin physiology and the relatively short observation period, potentially restricting the detection of long-term outcomes.

The potential instability of plant bioactives in hydrogel matrices may require future optimization using encapsulation techniques such as liposomal carriers or nanoemulsions. Additionally, hydrogels require more frequent applications than oleogels, and some formulations may lack transparency, making wound assessment more difficult [29].

No inflammatory biomarker profiling was performed in our study due to a lack of sufficient funding. Future work should include cytokine profiling (TNF-α, IL-6, VEGF) of the treated scald wounds to correlate the formulation efficacy with the inflammation response. The physicochemical and rheological characterization of all topical formulations using HPLC is currently in press and will provide further insight into the relationships between extract composition and the observed therapeutic responses. While no adverse effects were registered during cytotoxicity tests on cell cultures, further comparative analyses with variable extract concentrations would be valuable to refine the formulation.

Despite these challenges, the incorporation of bioactive plant extracts into hydrogel and oleogel formulations presents a promising avenue for burn wound management. Further clinical studies are warranted to confirm these preclinical findings, assess long-term healing outcomes, and evaluate patient-centric parameters such as pain relief, infection prevention, and cosmetic satisfaction. Clinically, these findings highlight oleogel formulations enriched with *Boswellia serrata* and *Ocimum basilicum* extracts as promising candidates for treating second-degree burns and could serve as a basis in clinical trials for the development of new local topical treatment options.

## 5. Conclusions

Oleogel-based topical formulations enriched with specific plant extracts demonstrate superior therapeutic efficacy in second-degree burn wound healing compared to hydrogel formulations, emphasizing their potential clinical utility and justifying further investigation in clinical settings. Specifically, formulations containing *Boswellia serrata* and *Ocimum basilicum* extracts significantly reduced wound size and inflammation, improved skin hydration, and decreased melanin production by days 9 and 21. While traditional treatments for second-degree burns remain foundational in clinical practice, the development of hydrogels and oleogels incorporating medicinal plant extracts offers promising ways of enhancing burn care. These innovative approaches aim to combine the benefits of advanced wound dressings with the therapeutic properties of natural compounds, improving healing outcomes and patient comfort. Future studies should explore human clinical trials and investigate the long-term stability of bioactive compounds in gel formulations under various storage conditions.

## Figures and Tables

**Table 1:** Overview of all topical formulations, detailing their type, extraction methods, active extracts with concentrations (*w*/*w*), gel matrix components, unique identifiers, and brief descriptions of key properties or intended functions.

Formulation	Extraction Method	Extract and Concentration (*w*/*w*)	Gel Components	Unique Name	Description
OG	ETOH99.5	OB 5%	Sunflower oil 80% Glyceryl dibehenate 15%	OG_OB_ETOH99.5	Oleogel with basil extract in absolute ethanol, based on glyceryl dibehenate and sunflower oil
OG	ETOH99.5	BS 5%	Sunflower oil 80% Glyceryl dibehenate 15%	OG_BS_ETOH99.5	Oleogel with frankincense extract in absolute ethanol, based on glyceryl dibehenate and sunflower oil
OG	ETOH99.5	SNF 5%	Olive oil 80% Glyceryl dibehenate 15%	OG_SNF_ETOH99.5	Oleogel with elderflower extract in absolute ethanol, based on glyceryl dibehenate and olive oil
OG	ETOH99.5	SNB 5%	Sunflower oil 80% Glyceryl dibehenate 15%	OG_SNB_ETOH99.5	Oleogel with elder bark extract in absolute ethanol, based on glyceryl dibehenate and sunflower oil
OG	ETOH99.5	GV 5%	Olive oil 80% Glyceryl dibehenate 15%	OG_GV_ETOH99.5	Oleogel with *Galium verum* extract in absolute ethanol, based on glyceryl dibehenate and olive oil
OG	ETOH70	OB 5%	Isopropyl myristate 80% Glyceryl dibehenate 15%	OG_OB_ETOH70	Oleogel with basil extract in 70% ethanol, based on glyceryl dibehenate and isopropyl myristate
OG	ETOH70	BS 5%	Sunflower oil 80% Glyceryl dibehenate 15%	OG_BS_ETOH70	Oleogel with frankincense extract in 70% ethanol, based on glyceryl dibehenate and sunflower oil
OG	ETOH70	SNF 5%	Diethylene glycol monoethyl ether 80% Glyceryl dibehenate 15%	OG_SNF_ETOH70	Oleogel with elderflower extract in 70% ethanol, based on glyceryl dibehenate and diethylene glycol monoethyl ether
OG	ETOH99.5	BS_OB_SNF_GV 1.25:1.25:1.25:1.25%	Sunflower oil 20% Olive oil 20% Isopropyl myristate 20% Diethylene glycol monoethyl ether 20% Glyceryl dibehenate 15%	OG_BS_OB_SNF_GV_ETOH99.5	Oleogel with 4 plant extracts in absolute ethanol
OG	ETOH70	BS_OB_SNF_GV 1.25:1.25:1.25:1.25%	Sunflower oil 20% Olive oil 20% Isopropyl myristate 20% Diethylene glycol monoethyl ether 20% Glyceryl dibehenate 15%	OG_BS_OB_SNF_GV_ETOH70	Oleogel with 4 plant extracts in 70% ethanol
HG	ETOH99.5	OB 5%	Purified water 60% Poloxamer 407 25% Glycerol 10%	HG_OB_ETOH99.5	Hydrogel with basil extract in absolute ethanol
HG	ETOH99.5	BS 5%	Purified water 60% Poloxamer 407 25% Glycerol 10%	HG_BS_ETOH99.5	Hydrogel with frankincense extract in absolute ethanol
HG	ETOH99.5	SNF 5%	Purified water 60% Poloxamer 407 25% Glycerol 10%	HG_SNF_ETOH99.5	Hydrogel with elderflower extract in absolute ethanol
HG	ETOH99.5	GV 5%	Purified water 60% Poloxamer 407 25% Glycerol 10%	HG_GV_ETOH99.5	Hydrogel with *Galium verum* extract in absolute ethanol
HG	ETOH70	OB 5%	Purified water 60% Poloxamer 407 25% Glycerol 10%	HG_OB_ETOH70	Hydrogel with basil extract in 70% ethanol
HG	ETOH70	BS 5%	Purified water 60% Poloxamer 407 25% Glycerol 10%	HG_BS_ETOH70	Hydrogel with frankincense extract in 70% ethanol
HG	ETOH70	SNF 5%	Purified water 60% Poloxamer 407 25% Glycerol 10%	HG_SNF_ETOH70	Hydrogel with elderflower extract in 70% ethanol
HG	ETOH99.5	SNB 5%	Purified water 60% Poloxamer 407 25% Glycerol 10%	HG_SNB_ETOH99.5	Hydrogel with elder bark extract in absolute ethanol
HG	ETOH70	BS_OB_SNF_GV 1.25:1.25:1.25:1.25%	Purified water 60% Poloxamer 407 25% Glycerol 10%	HG_BS_OB_SNF_GV_ETOH70	Hydrogel with 4 plant extracts in 70% ethanol
HG	ETOH99.5	BS_OB_SNF_GV 1.25:1.25:1.25:1.25%	Purified water 60% Poloxamer 407 25% Glycerol 10%	HG_BS_OB_SNF_GV_ETOH99.5	Hydrogel with 4 plant extracts in absolute ethanol
Pre-burn	N/A	Baseline	N/A	Pre-burn_baseline	Control for the healthy skin
Untreated_ Burn	N/A	Untreated_Burn	N/A	Untreated_burn_control	Control for the untreated burn
OG	N/A	OG_base	Sunflower oil 21.5% Olive oil 21.5% Isopropyl myristate 21.5% Diethylene glycol monoethyl ether 21.5% Glyceryl dibehenate 15%	OG_base	Control oleogel (without bioactive component)
HG	N/A	HG_base	Purified water 65% Poloxamer 407 25% Glycerol 10%	HG_base	Control hydrogel (without bioactive component), based on Poloxamer 407 and glycerol, purified water

**Table 2:** Overview of relevant studies examining plant-based topical formulations for wound healing. Columns specify the plant used along with the extract concentration, the solvent used for extraction, type of formulation, details on the base gel component and its concentration, the wound induction method employed, key outcomes assessed, and observations on the inflammatory response.

Paper	Plant (Extract Concentration)	Solvent Used	Formulation	Base Gel Component and Concentrations (*w*/*w*)	Wound Induction Method	Outcomes	Inflammatory Response
Mallik et al., 2010 [31]	*Boswellia serrata* (5%, 10%, 15%)	Ethanol	Cream	Base (N/A%)	In vivo Excision wound model	*Boswellia serrata* 15% *w*/*w* extract showed superior wound healing compared to control group. Wound contraction day 16: 98.02% vs. 64.13% in control.	5% *Boswellia serrata*: Moderate inflammation reduction. Day 8: 10% and 15% formulations—significant inflammatory marker inhibition. Day 12: 15% formulation nearly eliminated inflammatory response. Day 16: 15% formulation had 98.02% wound contraction, complete resolution of inflammation.
Antonescu et al., 2021 [23]	*Ocimum basilicum* (5%) *Trifolium pratense* (5%)	Hydro- alcoholic 70%	Hydrogel	Distilled water 78.0% Ethanol 10.0% Glycerin 10.0% Carbopol 940 1.0% Triethanolamine 1.0%	In vivo Burn wound model with metallic device	Days 13–16: Near-complete wound closure (100%) in hydrogel group; control had slower healing and incomplete remodeling.	EOT-based hydrogel—significant reduction in inflammation vs. control group. Day 13—complete wound healing in EOT gel group vs. partial healing in control.
Vuletic et al., 2022 [47]	*Galium verum* (20%)	Ethanol 70%	Mucoadhesive oral gel	Triethanolamine 0.7% Carbomer 934 (N/A%) Propylene glycol (N/A%) Sodium benzoate (N/A%) Purified water (N/A%)	In vivo Glacial acetic acid for oral ulcer	Days 3 and 6: Marked increase in collagen deposition, improved tissue remodeling. Day 6: Significantly lower COX-2 expression. Day 10: Complete ulcer closure with remodeled connective tissue.	GVL Gel—significantly higher wound contraction across all time points compared to control. Day 13—GVL Gel 95.1% healing vs. 81% in control
Mota et al., 2020 [44]	*Sambucus nigra* flower extract	Methanol	1. Placebo Gel 2. Free Plant Extract (0.15%) 3. PLGA Nanoparticle-Loaded Plant Extract (0.21%) 4. PCL Nanoparticle-Loaded Plant Extract (0.165%)	Carbopol 940 0.5% Methyl-4-hydroxybenzoate 0.2% Propyl-4-hydroxybenzoate 0.02% NaOH 0.2% Distilled water QS	In vivo Carrageenan-induced paw edema model	Moderate inflammation, slightly reduced edema compared to the untreated group. Mild fibroblast activation. Healing slightly better than control, no statistical significance.	41.2% paw edema reduction. Reduced inflammation, less strong effect compared to nanoformulated extracts or diclofenac.
Ali Khan et al., 2020 [29]	*Ocimum basilicum* leaves (5%)	Methanol 70%	Emulgel (5%)	Carbopol-934: 0.25%; Triethanolamine (TEA) Tween 80 0.30% Span 80 0.45% Liquid paraffin 3.75 Propylene glycol: 3.50 Methyl paraben: 0.01% Distilled water 50%	In vivo Carrageenan-induced paw edema model	Day 4: Severe inflammation, necrosis present. Days 8–12: Reduced inflammation, fibroblast proliferation, collagen remodeling. Day 16: Complete re-epithelialization, minimal inflammation, organized tissue.	75% wound contraction at day 8 with emuglel, similar to SSD; 25–30% lower TNF-α, IL-6 vs. untreated group. Reduced inflammation, enhanced fibroblast proliferation, and improved collagen deposition.
Dubey et al., 2017 [30]	*Ocimum basilicum* aerial parts (5%)	Hydro- alcoholic	Ointment	Lipophilic base ointment (N/A%)	In vivo Carrageenan-induced paw edema model	Day 8: Inflammation decreased, and fibroblast activation began in the extract-treated group. Day 12: Collagen deposition increased, while the control still showed inflammation. Day 16: The extract-treated wounds had organized collagen fibers and complete epithelialization, whereas healing was slower in the control group.	Extract-based cream significantly reduced inflammation vs. control (simple vehicle). Faster wound contraction (97.97% vs. 88.17% control), shorter epithelialization (18 vs. 22 days control), lower inflammatory infiltrates in study group. Plant extract modulates the inflammatory response, accelerating tissue regeneration.

## Data Availability

The original contributions presented in this study are included in the article. Further inquiries can be directed to the corresponding author.

## Abbreviations

The following abbreviations are used in this manuscript:

OB	*Ocimum basilicum*
BS	*Boswellia serrata*
GV	*Galium verum*
SNF	*Sambucus nigra* flower
SNB	*Sambucus nigra* bark
OG	Oleogel
HG	Hydrogel
SSD	Silver sulfadiazine
RAPID-3D	Rat Printed Induction Device—3D
PVP-I	Liposome polyvinyl-pyrrolidone-iodine
TA	Topical antimicrobial
RCT	Randomized controlled trial
